# When Aging Reaches CD4+ T-Cells: Phenotypic and Functional Changes

**DOI:** 10.3389/fimmu.2013.00107

**Published:** 2013-05-10

**Authors:** Marco Antonio Moro-García, Rebeca Alonso-Arias, Carlos López-Larrea

**Affiliations:** ^1^Immunology Department, Hospital Universitario Central de AsturiasOviedo, Spain; ^2^Fundación Renal “Iñigo Alvarez de Toledo”Madrid, Spain

**Keywords:** immunosenescence, T-cells, IL-15, inflammation, CMV, NKRs

## Abstract

Beyond midlife, the immune system shows aging features and its defensive capability becomes impaired, by a process known as immunosenescence that involves many changes in the innate and adaptive responses. Innate immunity seems to be better preserved globally, while the adaptive immune response exhibits profound age-dependent modifications. Elderly people display a decline in numbers of naïve T-cells in peripheral blood and lymphoid tissues, while, in contrast, their proportion of highly differentiated effector and memory T-cells, such as the CD28^null^ T-cells, increases markedly. Naïve and memory CD4+ T-cells constitute a highly dynamic system with constant homeostatic and antigen-driven proliferation, influx, and loss of T-cells. Thymic activity dwindles with age and essentially ceases in the later decades of life, severely constraining the generation of new T-cells. Homeostatic control mechanisms are very effective at maintaining a large and diverse subset of naïve CD4+ T-cells throughout life, but although later than in CD8 + T-cell compartment, these mechanisms ultimately fail with age.

## Introduction

Throughout life the aging of the immune system causes impairment of its defense capability, in a process known as immunosenescence. The aging process seems to alter the two branches of the immune system, the innate and the adaptive, in different ways. While the adaptive immune response undergoes profound age-dependent modifications (Haynes and Maue, 2009), innate immunity seems to be better preserved globally (Dace and Apte, 2008; Le Garff-Tavernier et al., 2010). The thymus, the development site of T-cells, atrophies with age (Dorshkind et al., 2009), with a direct impact on the proportions of naïve and memory T-cells. In aged animals and humans, the frequency of naïve CD4+ T-cells decreases, whereas the frequency of memory CD4+ T-cells increases (Nikolich-Zugich, 2005). Naïve and memory CD4+ T-cells are clearly distinct populations with unique cellular characteristics. Thus, any age-associated changes in CD4+ T-cell function including proliferation and cytokine production could be secondary to the alteration in the frequency of naïve and memory T-cells.

Despite CD4+ T-cells are more resistant to age-related phenotypic and functional changes than CD8+ T-cells (Weinberger et al., 2007), a progressive increase in the percentage of CD4+ T-cells that lack CD28 expression is common with increasing age in healthy individuals (Goronzy et al., 2007; Czesnikiewicz-Guzik et al., 2008) and in patients with chronic infections and autoimmune diseases (Fletcher et al., 2005; Thewissen et al., 2007). The accumulation of CD4 + CD28^null^ T-cells is partially explained by their reduced susceptibility to apoptosis and their oligoclonal expansions against *Cytomegalovirus* (CMV) and other chronic antigens (Almanzar et al., 2005; Pawelec and Derhovanessian, 2010). Loss of CD28 expression is a hallmark of the age-associated decline of CD4+ T-cell function. CD28 plays pivotal roles during T-cell activation, such as inducing cytokine production (IL-2) and promoting cell proliferation, so the lack of this costimulatory signal during activation results in a partial activation or even an anergic state of T-cells (Godlove et al., 2007). In this way, the accumulation of CD28^null^ T-cells is associated with a reduced overall immune response to pathogens and vaccines in the elderly (Saurwein-Teissl et al., 2002). In this way, CD4 + CD28^null^ T-cells can comprise up to 50% of the total CD4+ T-cell compartment in some individuals older than 65 years (Vallejo et al., 2000). CD4 + CD28^null^ T-cells acquire expression of several receptors commonly associated with natural killer (NK) cells, secrete large amounts of IFN-γ, and express perforin and granzyme B, which confer a cytotoxic capability on the cells (Appay et al., 2002b; van Leeuwen et al., 2004).

## CD4+ T-Cell Differentiation

Naïve CD4+ T-cells are activated after interaction with the antigen–major histocompatibility complex (MHC) complex and differentiate into specific subtypes depending mainly on the cytokine milieu of the microenvironment. The CD4+ T-cells carry out multiple functions, including activation of the cells of the innate immune system, B-lymphocytes, cytotoxic T-cells, as well as non-immune cells, and also play a critical role in suppressing the immune reaction. With the advent of multiparameter flow cytometry, it has become clear that individual cells can produce effector cytokines in different combinations (Seder et al., 2008), raising the question of whether there is heterogeneity within a lineage or whether each distinct cytokine combination represents a separate lineage. Continuing studies have identified new subsets of CD4+ T-cells besides the classical T-helper 1 (Th1) and T-helper 2 (Th2) cells. These include T-helper 17 (Th17), T-helper type 22 (Th22), follicular helper T-cell (Tfh), induced T-regulatory cells (iTreg), and the regulatory type 1 cells (Tr1) as well as the potentially distinct T-helper 9 (Th9). The differentiation of the various lineages depends on the complex network of specific cytokine signaling and transcription factors followed by epigenetic modifications.

**Figure 1:**
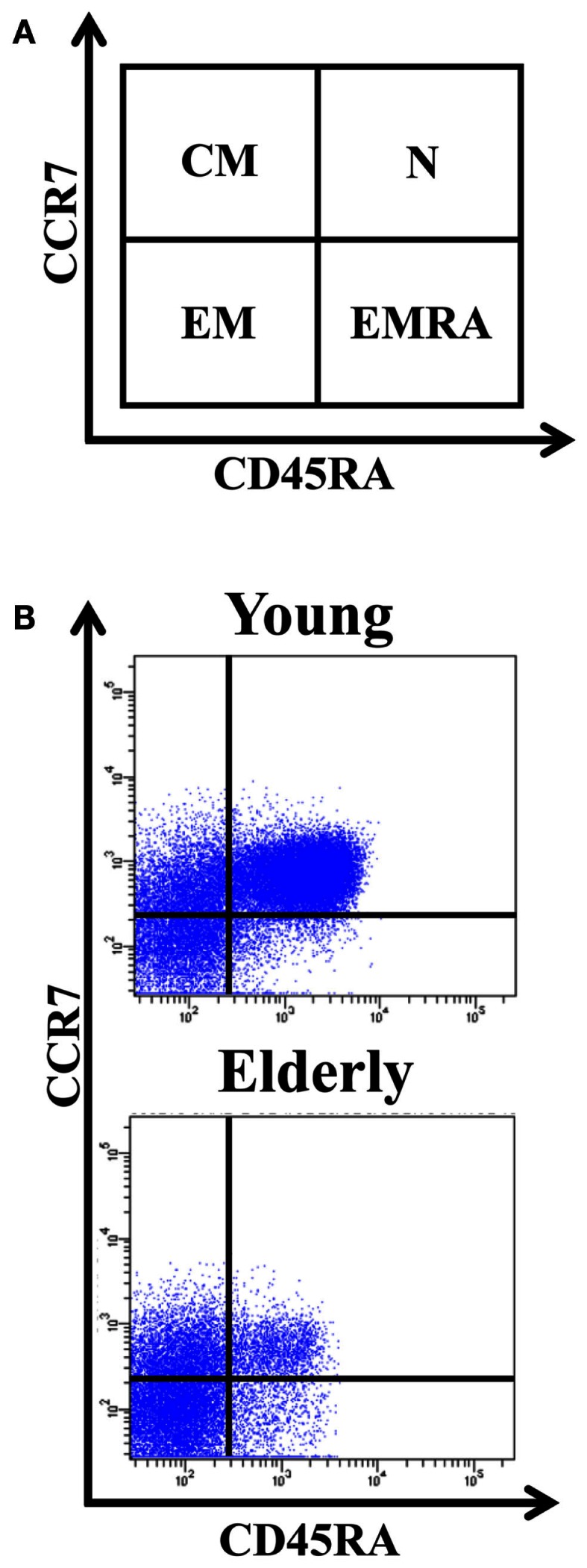
**Distribution of CD4+ T-cells into naïve, central memory, effector memory (EM), and effector memory RA (EMRA)**. **(A)** Schematic model of the T-cells differentiation subsets accordingly to CD4 + 5RA and CCR7 expression. **(B)** Dot-plots representatives of the naïve, CM, EM, and EMRA subsets in young people and elderly subjects into the CD4+ T-cells. Whole blood was stained with anti-CD45RA-FICT, anti-CD8-PE, anti-CD4-PerCP, and anti-CCR7-APC, and 10^5^ cells were acquired in each experiment.

**Figure 2:**
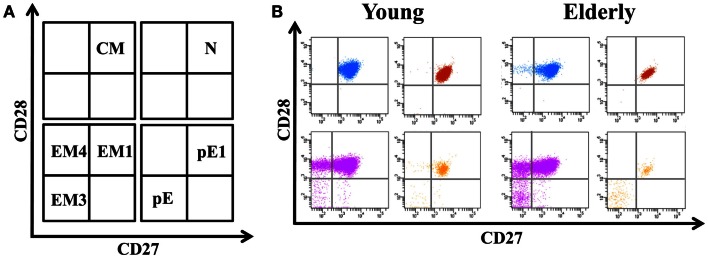
**Distribution of EM and EMRA in CD4+ T-cells into subsets defined by CD28 and CD27 expression**. **(A)** Schematic model of the EM and EMRA CD4+ T-cells differentiation subsets accordingly to CD27 and CD28 expression. EM T-cells can be divided into EM1 (CD27 + CD28 +), EM2 (CD27 + CD28^null^, only in CD8 +  T-cells), EM3 (CD27^null^CD28^null^), and EM4 (CD27^null^CD28 +). Similarly, EMRA can be divided into pE1 (CD27 + CD28+) and pE2 (CD27 + CD28^null^, only in CD8 T-cells) and E (CD27^null^CD28^null^). **(B)** Dot-plots representatives of the EM and EMRA subsets in young people and elderly subjects into the CD4+ T-cells.

The differentiation of naïve CD4+ T-cells into effector and memory subsets is one of the most fundamental facets of T-cell-mediated immunity. CD4+ T-cells can be separated into functionally distinct populations using combinations of cell surface markers, such as the tyrosine phosphatase isoform CD45RA+ and the chemokine receptor CCR7 (Figure 1). With these markers, we subdivided the T-cells into naïve (NAÏVE; CD45RA + CCR7+), central memory (CM; CD45RA − CCR7+), effector memory (EM; CD45RA − CCR7-), and effector memory RA+ (EMRA; CD45RA + CCR7-) cells (Sallusto et al., 1999). EM is a heterogeneous population, and the staining of two additional markers, CD27 and CD28, has proved useful for identifying the less differentiated EM1 (CD28+ and CD27+) and EM4 (CD28+ and CD27^null^) subsets, and the more differentiated EM3 cells (CD27^null^CD28^null^) (Figure 2). The EMRA subset can be further subdivided into very poorly differentiated pE1 (CD27 + CD28 +) and the most highly differentiated T-cell subset, E (CD27^null^CD28^null^) (Koch et al., 2008) (Figure 2). Differentiating CD4+ T-cells lose expression of CD27 first, then of CD28 in a later phase (Amyes et al., 2003; van Leeuwen et al., 2004). In contrast, CD8+ T-cells lose expression of CD28 first and then of CD27 (Gamadia et al., 2003).

### Naïve CD4+ T-cells

Naïve T-cells are characterized by the expression of surface markers CD45RA, CD27, CD28, CD62L, CCR7, and the IL-7 receptor (De Rosa et al., 2001; Swainson et al., 2006). Naïve T-cells exit the thymus following maturation and are enriched for T-cell receptor excision circles (TREC) and express the surface marker CD31 (Kimmig et al., 2002). Naïve T-cells circulate between the blood and the lymphoid tissue driven by cell surface markers CD62L and CCR7 (Sallusto et al., 1999). The number of naïve T-cells in the blood remains relatively constant throughout adult life despite continuous stimulation with foreign antigens and a dramatic reduction in thymic output with age. Although *thymic involution* is a well-known phenomenon, no satisfactory explanation for its existence has been offered (Lynch et al., 2009). Several hypotheses have argued that this age-related change is adaptive rather than detrimental (Aronson, 1991; O’Leary and Hallgren, 1991; Dowling and Hodgkin, 2009). Accordingly, thymic involution may represent a mechanism for how the body is able to achieve the remarkable balancing act of avoiding autoimmunity and maintaining a sufficiently diverse repertoire to combat a large number of potential pathogens. Some possible causes of thymic involution may be the blocking of the rearrangement of T-cell receptor (TCR) genes (Aspinall, 1997), self-peptide MHC-decreased molecules (Lacorazza et al., 1999), and loss of T-cell progenitors (Zoller et al., 2007). The importance of the thymus for developing adequate cellular immunity can be studied in the context of several disease states (associated with thymic ablation or hypoplasia). Young people who were thymectomized within 2 weeks of birth display several immunological alterations, including lower CD4+ or CD8 +  T-cell counts, reduced proportions of recent thymic emigrants and naïve cells, accumulation of oligoclonal memory T-cell populations, and increased levels of inflammation markers (Sauce et al., 2009; Zlamy and Prelog, 2009).

Exposure to the cytokine IL-7 and contact with MHC molecules presenting self-peptides through the TCR within secondary lymphoid tissue are both essential for naïve T-cell homeostasis (Brocker, 1997; Tan et al., 2001; den Braber et al., 2012). When these naïve T-cells do encounter antigens on activated dendritic cells (DCs) in central lymphoid organs, they proliferate and differentiate into effector T-cells. When the antigen has been cleared, a contraction phase follows, during which time the number of effector cells declines through apoptosis, leaving behind some survivors that go on to differentiate into memory T-cells.

### Central memory

Human CM are CD45R0+ memory cells that constitutively express CCR7 and CD62L, two receptors that are also characteristic of naïve T-cells, and which are required for cell extravasation through high endothelial venules (HEV) and migration to T-cell areas of secondary lymphoid organs (Campbell et al., 1998; Forster et al., 1999). Homeostatic proliferation ensures the longevity of CM T-cells by inducing cell proliferation in the absence of cellular differentiation or activation. This process is governed mainly by IL-7. Nonetheless, CM T-cells can also be stimulated via engagement of the TCR, leading to proliferation but also activation and differentiation (Bosque et al., 2011). Compared with naïve T-cells, CM have higher sensitivity to antigenic stimulation, are less dependent on costimulation, and upregulate CD40L to a greater extent, thus providing more effective stimulatory feedback to DCs and B cells. Following TCR triggering, CM produce mainly IL-2, but after proliferation they efficiently differentiate into effector cells and produce large amounts of IFN-γ or IL-4.

### Effector memory

Human EM are memory cells that have lost the constitutive expression of CCR7, are heterogeneous for CD62L expression, and display characteristic sets of chemokine receptors and adhesion molecules that are required for homing to inflamed tissues. Compared with CM, EM cells are characterized by a rapid effector function. They produce IFN-γ, IL-4, and IL-5 within hours of antigenic stimulation. The relative proportions of CM and EM in blood vary in the CD4+ and CD8+ T-cells. CM is predominant in CD4+ and EM in CD8+ (Taylor and Jenkins, 2011). Within the tissues, CM cells are enriched in lymph nodes and tonsils, whereas lung, liver, and gut contain greater proportions of EM (Campbell et al., 2001). Increasing evidence indicates the existence of highly heterogeneous functional EM subpopulations: EM1, which is very similar to EM4, and EM3. EM1 and EM4 are memory-like, and EM3 is effector-like. Taken together, these data are consistent with the model according to which there is a differentiation pathway with progressive loss of CCR7, CD27, and CD28 cell surface expression concomitant with upregulation of cytolytic capacity (Appay et al., 2002a).

### Effector memory RA

Persistent viral infections and inflammatory syndromes induce the accumulation of T-cells with characteristics of terminal differentiation or senescence. However, the mechanism that regulates the end-stage differentiation of these cells is unclear. EMRA T-cells have features of telomere-independent senescence that are regulated by active cell signaling pathways that are reversible. These EM T-cells that re-express CD45RA (CCR7-CD45RA+; EMRA) have many characteristics of end-stage differentiation. The EMRA subset can be further subdivided into very poorly differentiated pE1 (CD27 + CD28+) and the most differentiated T-cell subset, E (CD27^null^CD28^null^). However, the exact nature of these T-cells is not clear.

## Phenotypic and Functional Changes Associated with Aging

As we age, the CD4+ T-cells are repetitively stimulated by a large number of different antigens and as a consequence, CD4+ T-cells become refractory to telomerase induction, suffer telomere erosion, and enter replicative senescence. Replicative senescence is characterized by the accumulation of highly differentiated T-cells with newly acquired functional capabilities, which can be caused by aberrant expression of genes normally suppressed by epigenetic mechanisms in CD4+ T-cells. Age-dependent demethylation and overexpression of genes normally suppressed by DNA methylation have been demonstrated in senescent subsets of T-lymphocytes (Lu et al., 2003; Liu et al., 2009). There are some major features of CD4+ T-cell that are acquired as they age: loss of proliferative capacity and telomerase activity, TCR restriction, low production of and response to IL-2, high response to IL-15, loss of expression of CD28 molecule, expression of NK cell-related receptors (NKRs), production of molecules involved in cellular cytotoxicity (perforin and granzyme), and a substantial increase in the production of IFN-γ (Appay et al., 2008) (Table 1).

**Table 1:** **Functional differences between naïve and late-memory CD4+ T-cells**.

Naïve	CD4+	Late-memory
↑↑↑	Proliferative capacity	↓↓
↑↑↑	Telomerase activity	↓↓
–	TCR restriction	↑↑
↑↑↑	IL-2 production and response	↓↓
–	Response to IL-15	↑↑
↑↑↑	CD28 expression	↓↓
–	NKRs	↑↑
↑↑	IFN-γ production	↑↑↑
–	Cellular cytotoxicity (perforin and granzyme)	↑↑

### The costimulatory molecule CD28 in aged CD4+ T-cell

Several studies have demonstrated that expression of TCR in CD4+ T-cells are not altered in the elderly (Bazdar et al., 2009), however, costimulatory molecules required for lymphocyte activation appear to be altered. One of the major costimulatory molecules present in T-lymphocytes is the CD28 molecule, and its loss as individuals age is well-documented in CD4+ T-cells (Weyand et al., 1998). Loss of CD28 has been associated with a loss of immune system responsiveness in the elderly. These cells are less able to proliferate than are CD4 + CD28+ T-cells, have a diminished antigenic recognition repertoire, and gain a very powerful cytotoxic capacity (Bryl and Witkowski, 2004). CD28 downregulation occurs with T-cell activation, involving transcriptional repression and increased protein turnover. This is thought to be a negative feedback mechanism (Swigut et al., 2001). When CD4+ T-cells recognize an antigen, CD28 expression decreases rapidly, but immediately returns to normal levels. However, with sustained stimulation over time, the expression of CD28 decreases and may be lost. CD28 can be initially reinduced by IL-12 (Warrington et al., 2003) or with treatment with anti-TNF agents (Rizzello et al., 2006), but once firmly established, its loss is irreversible in the majority of CD28^null^ T-cells, suggesting active transcriptional silencing.

### Acquisition of new aging markers

**Figure 3:**
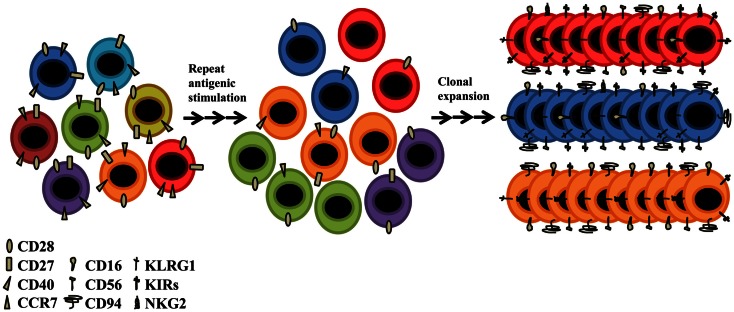
**Older people present a different receptor repertoire from that of young individuals**. Despite the decline of thymic function and the low TCR diversity, the elderly CD4+ T-cells present novel functions attributed to their acquisition of NK-related receptors (NKRs) such as KIR, CD94, CD16, CD56, NKG2, and KLRG1. CD4+ T-cells undergo senescence due to lifetime exposure to persistent pathogens and to homeostatic proliferation. With advancing age, the T-cell repertoire becomes populated with highly oligoclonal, long-lived T-cells, most of which have lost the ability to express CD28. Such CD28^null^ T-cells have limited proliferative capacity, but are functionally active. They are generally long-lived and functionally active.

Although CD28 is a major costimulatory molecule, these CD28^null^ T-cells remain functionally active; other molecules must be able to maintain responsiveness and survival in these cells. Therefore, alternative receptors must exist to prevent these cells entering into a state of anergy. CD4 + CD28^null^ T-cells are resistant to apoptosis (Vallejo et al., 2000), which is one possible cause of its accumulation throughout life (Posnett et al., 1994). An explanation of why these cells are able to be activated, is the *de novo* expression of several NKRs (Abedin et al., 2005). Among the best studied are the receptors CD16, CD56, CD94, KLRG1, several members of the NK receptor G2 (NKG2), and the killer cell immunoglobulin (Ig)-like receptor (KIR) families. CD94, KLRG1, and the NKG2s are lectin-like receptors, and CD16 and CD56 are receptors belonging to the superfamily of immunoglobulins, and are the prototypic NKRs that are normally used to identify NK cells (Figure 3). The functional roles of CD16, CD56, and CD94 on senescent T-cells are still unknown. The KLRG1 receptor seems to influence the state of CD4+ T-cell senescence due to their ability to inhibit proliferation via TCR (Hayhoe et al., 2010; Di Mitri et al., 2011). KLRG1 contains an immunoreceptor tyrosine-based inhibitory motif (ITIM) in its cytoplasmic domain and has been shown to be a receptor for some members of the cadherin family of proteins (Grundemann et al., 2006). It is an inhibitory receptor and its presence in CD4+ T-cells blocks the costimulatory activities mediated by Akt, such as proliferation (Henson et al., 2009). Among NKG2s receptors, only NKG2D is expressed in CD4 + CD28^null^ aged T-cells, its expression becoming present for the first time in CD4 + CD28^null^ T-lymphocytes as people age. This novel age-marker was recently described by our laboratory (Alonso-Arias et al., 2011b). It has been implicated in NK-mediated anti-viral immunity and in TCR-independent cytotoxic activity in CD4+ and CD8+ T-cells. The regulation of KIRs seems to differ in NK cells and T-lymphocytes (Xu et al., 2005). The KIR repertoire in T-cells is very restricted (Abedin et al., 2005), being limited to memory T-cells, mainly CD28^null^ T-lymphocytes. In addition, the same population of T-lymphocytes with the same TCR specificity may have different combinations of KIRs on their surface (Vely et al., 2001; van Bergen et al., 2009). It seems quite clear that the expression of NKRs differs in oligoclonal and senescent T-cells. The expression of these molecules appears to represent a different way of diversifying the immune repertoire, i.e., an oligoclonal population of T-lymphocytes for a particular TCR can express a wide diversity of receptor NKRs codominantly (Tarazona et al., 2000) (Figure 3). In the case of arterial disease and CMV infection, the expression of KIR receptors in CD4 + CD28^null^ T-cells is broadly accepted as being responsible for their functionality (Zal et al., 2008; van Bergen et al., 2009). The appearance of these “aberrant” molecules in senescent T-cells could help maintain the adequate homeostasis of T-cells and would be a way to stay functionally active, independent of TCR activation.

### Functional properties acquired by aging CD4+ T-cells

A defining feature of the eukaryotic genome is the presence of linear chromosomes. This arrangement, however, poses several challenges with regard to chromosomal replication and maintenance. Telomeric DNA is lost due to the incomplete terminal synthesis of the lagging DNA strand during cell division. Immune cells must be able to grow exponentially and die when no longer needed. They support an extremely high replicative rate, so their telomeres suffer great stress. The lymphocytes are capable of upregulating telomerase, an enzyme that elongates telomeres and can therefore prolong the life of the cell (Klapper et al., 2003; Andrews et al., 2010). Signaling via the TCR and other costimulatory molecules, such as CD28, are necessary for inducing telomerase activity with a peak of activation at 4–5 days after stimulation and a decrease in activity at 10 days (Macallan et al., 2004; Fritsch et al., 2005). In the absence of mechanisms that compensate for telomere shortening, growth arrest of the cells occurs when progressive telomere erosion reaches a critical point known as replicative senescence (Hodes et al., 2002). The overall finding from several different studies is that human T-cells can undergo a limited number of divisions, after which they cease dividing (Perillo et al., 1989). Importantly, the arrival of T-cells at a stage of replicative senescence does not imply the loss of cell viability. In fact, under appropriate conditions senescent cells remain alive and metabolically active for a long period (Wang et al., 1994). In cultures where CD4+ and CD8+ T-cells of the same subject are stimulated identically, CD8+ T-cells were unable to upregulate telomerase after the fourth encounter with the antigen. In contrast, the CD4+ T-cells from the same donor had a high level of telomerase activity induced by antigen (Valenzuela and Effros, 2002). Several studies have shown that telomerase activity is preserved and replicative senescence is delayed if telomere length is stabilized (Dagarag et al., 2004; Choi et al., 2008). The inhibition of cytokines involved in shortening telomeres, such as TNF-α, could delay telomeric loss. One of the main causes of cell division is the interaction of TCR and CD28 receptors that leads to the production of cytokines. One of the best studied is IL-2, which is produced in an autocrine form and that causes upregulation of its own receptor (IL-2R), composed of three subunits (α, β, and γ) (Almeida et al., 2002). The γ-chain is common to other cytokine receptors such as IL-7, IL-15, and IL-21, and the differences in the responses elicited by these cytokines must lie in the other two chains that form the receptor. The main molecules involved in signaling via IL-2 are Janus kinases (Jaks) and signal transducer and activator of transcription (STATs) (Johnston et al., 1996). One of the first indications that the immune system of the elderly has impaired functionality was the reduction in the production of IL-2 (Caruso et al., 1996). Several studies have demonstrated that levels of TCR in T-cells are not altered in the elderly, for which reason it is thought that the problem may be to do with intracellular signaling (Bazdar et al., 2009). Alterations in intracellular signaling may partly explain the lack of production of IL-2 in the elderly.

CD4+ T-cells have not been classically considered as cytotoxic cells, although intracytoplasmic stores of granzyme B and perforin have been previously described in CD4 + CD28^null^ T-cells (Appay et al., 2002b). Granzyme B and perforin expression in CD4+ T-cells are closely associated with the loss of CD28 from the cell surface. These CD4 + CD28^null^ T-cells resemble cytotoxic CD8+ T-cells, because their cytotoxic capacity is mediated by TCR stimulation. In addition, they lack costimulatory molecule requirements (Appay et al., 2002a). The expression of NK molecules described above is associated with increased cytotoxic capacity with high levels of expression of intracytoplasmic perforin and granzyme (Brown et al., 2012). The expression of these NK receptors in CD4+ T-cells probably serves to regulate their cytotoxicity, and even cytokines involved in NK cell activation, such as IL-15, can enhance their cytotoxic ability. The expansion of these cells not only occurs in the elderly, but also under other clinical conditions involving chronic activation of the immune system, such as viral infections, autoimmune and rheumatic diseases, certain tumors, and coronary artery disease (Thewissen et al., 2007; Alonso-Arias et al., 2009). CD4 + CD28^null^ T-cells also secrete large amounts of IFN-γ. CD4 + CD28^null^ T-lymphocytes have been described as being antigen-specific cells against chronic viral antigens, mainly in some autoimmune diseases (Thewissen et al., 2007). IFN-γ expression is present at all stages of CD4+ T-cell differentiation, but is mostly improved in late-differentiated cells that lack IL-2-production (Yue et al., 2004; Harari et al., 2005). The dominant IFN-γ CD4+ T-cell response is associated with models of antigen persistence and high antigen levels.

It has been hypothesized that CD4 + CD28^null^ T-cells might play a role in containing viral infections tropic for HLA class II cells, such as EBV in B cells, HIV-1 in activated CD4+ T-cells, monocytes and DCs, and CMV in endothelial cells. However, the presentation mechanism of this antigen is not currently known. In the case of CMV infection, endothelial cells are poor antigen-presenting cells under normal conditions in a classical immune response because they lack costimulatory molecules. Nevertheless, since the CD28^null^ T-cells do not require costimulation and have a low activation threshold, antigen presentation could be rendered effective by non-professional cells such as endothelial cells. This hypothesis is supported by the fact that the class II pathway may be preferentially targeted, since both EBV and CMV prevent normal MHC class I expression as part of their strategies of immune evasion (Alcami and Koszinowski, 2000).

## Immune Functional (Dis)Ability

The human immune system progressively deteriorates with age, leading to a greater incidence or the reactivation of infectious diseases, as well as to the development of autoimmune disorders and cancer (DelaRosa et al., 2006; Prelog, 2006). These defective immune responses are also manifested in a reduced capacity to induce immunological memory to vaccines and infections. In fact, the incidence of acute transplantation rejections is significantly lower in elderly transplant patients (Bradley, 2002; Deng et al., 2004; Trzonkowski et al., 2010). Immunological impairment may be partially due to the restriction of antigen recognition (Figure 3). Protection from pathogens and tumor development depends on the generation and maintenance of a diverse TCR repertoire. CD4+ and CD8 T-cells undergo the same principal phenotypic shifts; however, the rate at which they occur or accumulate with age is vastly different. Diminution of naïve cells with age is drastic for CD8 T-cells, but relatively minor for CD4+ T-cells. Homeostatic control of the CD4+ compartment is much more robust than that of CD8 T-cells. In spite of the majority of naïve T-cells in the adult being generated by IL-7- and IL-15-induced division of pre-existing cells, the diversity of the naïve CD4+ T-cell repertoire is maintained up to the age of 65 years (Prlic and Jameson, 2002; Naylor et al., 2005). At older ages, TCR diversity is remarkably reduced by accumulation of clonal cells in both naïve and memory compartments (Vallejo, 2007). Elderly donors display a marked increase in the proportion of highly differentiated effector and memory T-cells due to a lifetime of exposure to a variety of pathogens. Accumulation of these highly differentiated T-cells is partially explained by their reduced susceptibility to apoptosis and their oligoclonal expansions against CMV and other chronic antigens (Vescovini et al., 2004; Almanzar et al., 2005; Vasto et al., 2007; Derhovanessian et al., 2009). Persistent viral infections and/or the pro-inflammatory cytokines produced during some infectious processes may drive their differentiation. Another possible explanation is the corroborated fact that advanced age is accompanied by low-grade, chronic upregulation of inflammatory responses, evidence for which is provided by increased serum levels of pro-inflammatory cytokines (IL-6, IL-15, IL-8), coagulation factors, and reactive oxygen species (ROS) (Mari et al., 1995; Forsey et al., 2003; Zanni et al., 2003; Ferrucci et al., 2005; Wikby et al., 2006; Giunta et al., 2008). Since the number of circulating T-cells is maintained over the lifespan, a compensatory mechanism may give rise to an increase in highly differentiated memory cells in parallel with the reduction in naïve cell proliferation. Even the higher absolute counts of highly differentiated CD8+ T-cells could modulate the levels of CD4+ T-cells. Experienced T-lymphocytes, mainly CD8+, may fill the immunological space, and homeostatic mechanisms block the generation of new naïve cells to maintain the numbers of peripheral T-lymphocytes (Alonso-Arias et al., 2013). These mechanisms make it difficult to preserve the T-cell repertoire diversity that combats new pathogens and the host’s ability to mount vigorous recall responses to recurrent infections (Nikolich-Zugich, 2008). Another of the most prominent changes during T-cell aging in humans is the change in the functional ability of the T-cells with a high degree of differentiation. CD28 is pivotal in T-cell activation, doing such things as inducing cytokine production (IL-2) and promoting cell proliferation, so the lack of this costimulatory signal during activation results in a partial activation or even an anergic state of T-cells (Godlove et al., 2007). In contrast, CD4 + CD28^null^ T-cells have a low activation threshold, which could play a part in their predisposition to the breakage of self-tolerance (Yung et al., 1996). In this way, the accumulation of CD28^null^ T-cells, particularly within the CD8 subset, is associated with a reduced overall immune response to pathogens and vaccines in the elderly (Saurwein-Teissl et al., 2002; Alonso-Arias et al., 2013).

## Effect of IL-15 Homeostatic Cytokine on Highly Differentiated CD4+ T-Cells

**Figure 4:**
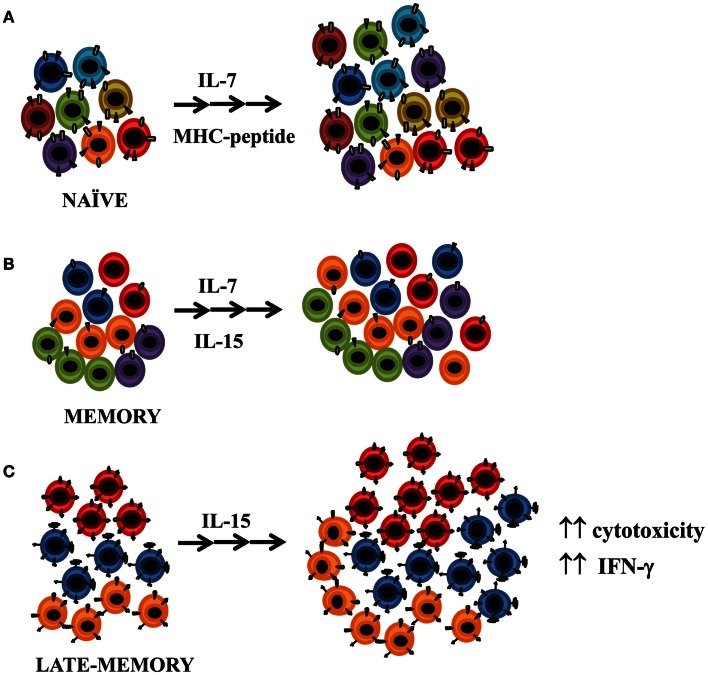
**Effect of IL-15 homeostatic cytokine on CD4+ T-cells**. **(A)** It is widely accepted that IL-7 signaling through the IL-7 receptor is essential for prolonged survival of naïve and memory T-cells. Naïve T-cells rely on survival signals through contact with self-peptide-loaded major histocompatibility complex (MHC) molecules plus interleukin IL-7. **(B)** Antigen-experienced (memory) T-cells are typically MHC-independent. They survive and undergo periodic homeostatic proliferation through contact with both IL-7 and IL-15. **(C)** IL-15 promotes the proliferation of late-memory CD4+ T-cells and enhances the proliferative response of CD28^null^ cells with respect to CD28+ CD4+ T-cells. IL-15 increases the cytolytic properties of CD4 + CD28^null^ T-cells and enhances their antigen-specific responses.

It is widely accepted that IL-7 signaling through the IL-7 receptor (IL-7R), is essential for prolonged survival and proliferation of naïve and memory T-cells. Naïve T-cells rely on survival signals through contact with self-peptide-loaded MHC molecules plus interleukin IL-7. On the other hand, antigen-experienced (memory) T-cells are typically MHC-independent and survive and undergo periodic homeostatic proliferation through contact with both IL-7 and IL-15 (Boyman et al., 2012) (Figure 4). Both cytokines seem equally essential to enable these cells to undergo basal homeostatic proliferation (Lenz et al., 2004; Purton et al., 2007), but IL-15 has a less prominent role for memory CD4+ cell homeostasis than for NK and memory CD8+ cells (Surh and Sprent, 2008). Memory CD4+ T-cells compete less effectively for IL-15 than the latter cells since they have much lower levels of expression of the IL-15 receptor (Lenz et al., 2004). Homeostatic proliferation of T-cells can be one cause of the age-associated loss of CD28 expression, since CD8+ memory T-cells in the presence of IL-15 alone, without TCR stimulation, lose CD28 expression and proliferate at a similar rate to CD8+CD28+ T-cells (Chiu et al., 2006). In contrast, IL-15 does not induce loss of CD28 expression in CD4+ T-cells, although recent studies have shown that IL-15 does in fact play an appreciable role in CD4+ memory T-cell proliferation under physiological conditions and after *in vitro* stimulation (Geginat et al., 2001; Lenz et al., 2004; Alonso-Arias et al., 2011b) (Figure 4). CD4+ memory T-cells rely on STAT5, the downstream signaling molecule used by IL-15, considerably more than do effector CD4+ T-cells (Purton et al., 2007; Tripathi et al., 2010). IL-15 increased the cytolytic properties of CD4 + CD28^null^ T-cells and enhanced their antigen-specific responses (Alonso-Arias et al., 2011b). Although the role of CD4+ T-cells as cytotoxic effector cells is not well understood, the enhancing effector activity of IL-15 may have a substantial impact, since CD4 + CD28^null^ T-cells are mainly specific against chronic contact antigens. Moreover, IL-15 plays a critical role in the immune responses to early infection and chronic inflammation by amplifying the effects of pro-inflammatory cytokines on IFN-γ secretion and by enhancing the antigen-specific responses of CD4 + CD28^null^ (Smeltz, 2007; Alonso-Arias et al., 2011b).

## Inflammation and CMV as Inductors of CD4+ T-Cell Aging

The degree of immunosenescence varies greatly, even among age-matched elderly individuals (Alonso-Arias et al., 2011a). This may mean that individual or environmental factors influence immunological status in different ways. In younger individuals, the inflammatory response is necessary to protect against infectious and damaging agents, but it can be detrimental in later life (Franceschi et al., 2007). As a result of continual antigenic stress throughout life, chronic low-grade inflammation develops, and this is considered to be a major contributor to age-associated frailty, morbidity, and mortality (Franceschi et al., 2000). Progressive T-cell differentiation and low-grade inflammation are two processes that occur simultaneously and/or enhance each other. Highly differentiated cells help increase the levels of pro-inflammatory cytokines, whereas inflammatory mediators are involved in the development of differentiated T-cell phenotypes. The ability to prevent or block this inflammatory status may be responsible for the differences seen between individuals. In centenarians, who are commonly considered a paradigm of “successful aging,” the chronic pro-inflammatory state of aging is countered by increased expression of anti-inflammatory cytokines. In this way, the frequency of the IL-10 (−1082GG) genotype, associated with increased production of this anti-inflammatory cytokine, is higher in centenarians than in younger controls (Lio et al., 2004). In parallel, their immune system exhibits no signs of a T-cell Immune Risk Profile (IRP), comprising a group of immune parameters which has been defined as an inverted CD4/CD8 ratio, an accumulation of CD8 + CD28^null^ T-cells, and CMV infection (Olsson et al., 2000). The inverted CD4/CD8 ratio was the sole marker significantly associated with the IRP. Subsequently, CMV infection has been shown to exert a major impact on the immunosenescence process (Hadrup et al., 2006). The Swedish OCTO and NONA immune longitudinal studies were able to identify and confirm the IRP predictive of an increased 2-year mortality in very old individuals, 86–94 years of age. Recently, a similar study conducted in subjects aged 66, indicates that the IRP could be also associated with increased mortality in hexagenerians. Therefore, it will be important to examine morbidity and mortality to assess whether the immune profile also is an IRP in the hexagenerians (Strindhall et al., 2012).

One of the main factors affecting longevity could be represented by a well-functioning immune system that prevents the main age-related chronic diseases such as atherosclerosis, type 2 diabetes, and Alzheimer’s disease (Pradhan et al., 2001; Libby et al., 2002; Griffin, 2006). Even depression and frailty (the latter an emerging clinical entity occurring late in life), which are correlated with increased morbidity and mortality within a few years, have a major inflammatory component (De Martinis et al., 2006; Raison et al., 2006). These pathologies are all also characterized by important alterations in the CD4+ T-cell compartment, resulting in lower proportions of naïve cells and higher proportions of late-differentiated cells (Dumitriu et al., 2009; Giubilato et al., 2011; Pellicano et al., 2012). Recently, CMV has been linked to this range of chronic diseases with an inflammatory component (Harkins et al., 2002; Aiello et al., 2006; Simanek et al., 2009; Moro-Garcia et al., 2012). The specific mechanisms responsible for these associations are not fully determined but are likely to have an inflammatory and immune component. After infection, the virus establishes lifelong latency within the host and periodically reactivates. Reactivation from latency is a key step in the pathogenesis of the infection and can be detected in response to inflammation, infection, stress, or immunosuppression (Kutza et al., 1998; Prosch et al., 2000). Activation of protein kinase C and NF-κB by TNF-α and increasing concentrations of cyclic AMP by stress hormones and prostaglandins promotes viral reactivation and replication. Reactivation of CMV is more frequent in the elderly and the virus, in turn, may result in increased levels of pro-inflammatory molecules such as IL-6, TNF-α, and C-reactive protein (CRP) (Stowe et al., 2007), contributing to the increase in the inflammatory status. These more frequent and/or intense reactivations in the elderly may be a consequence rather than the cause of immunosenescence. Furthermore, reactivations imply repetitive reencounters between specific T-cells and CMV antigens, leading to their activation and proliferation and consequent aging.

Despite the evidence suggesting that CMV induces aging of T-lymphocytes, more frequent and/or intensive reactivations in the elderly may be a consequence rather than the cause of immunosenescence. CMV seropositivity and anti-CMV antibody titers are related to the degree of differentiation of CD4+ T-cells and to the other IRP parameters of elderly people (Olsson et al., 2000; Alonso-Arias et al., 2013). Differences between elderly and young individuals in highly differentiated and naïve CD4+ T-cells become more marked, depending on their anti-CMV antibody titers.

Strategies directed at counteracting the inflammatory status in the elderly have been evaluated. Cross-sectional studies reveal an association between physical inactivity and low-grade systemic inflammation in elderly people (Wannamethee et al., 2002; King et al., 2003). Sedentary elderly individuals have a greater risk of mortality than those doing intermediate or high levels of physical activity, who have a reduced risk of coronary heart disease, neurodegeneration, cancer incidence, and disability (functional impairment) (Hambrecht et al., 2000; Melzer et al., 2004; Lautenschlager et al., 2011; Speelman et al., 2011). Elderly individuals with functional disability, which implies mobility impairment, even to the point of not being able to perform all daily activities adequately, also have smaller naïve CD4+ T-cell subpopulations and higher percentages of effector cells, together with reduced anti-CD3 responses. However, their responses to CMV gradually increase. The underlying mechanisms conferring protection are not known but it is thought that the anti-inflammatory role of moderate physical activity may be an influence (Pedersen and Saltin, 2006; Walsh et al., 2011; Warren et al., 2011). This anti-inflammatory effect of exercise may be responsible for the beneficial effects of exercise on health, and may play important roles in the protection against aging of the immune response and diseases associated with low-grade inflammation.

## Concluding Remarks

Changes similar to those observed in CD8+ T-cells during aging appear, albeit belatedly, in the compartment of CD4+ T-lymphocytes. These aged CD4+ T-cells can be found in the elderly and in individuals under inflammatory and/or antigenic stress due to autoimmune or chronic infectious processes. All these events in CD4+ T-cells appear at late stages in life, correlating with the impaired health status in elderly people. This impairment may be the result of their restricted immune response, as reflected by their reduced ability to fight against pathogens and poorer response to vaccination. A possible field of action to prevent the deterioration of the adaptive immune response would be the “rejuvenation” of the CD4+ T-cell population. Preclinical and clinical studies on the T-cell reconstitution effects of sex steroid ablation, keratinocyte growth factor, the growth hormone pathway, and the cytokines IL-7, IL-12, and IL-15 indicate that these strategies may be used to alleviate the effects of T-cell deficiencies in the aging immune system.

## Conflict of Interest Statement

The authors declare that the research was conducted in the absence of any commercial or financial relationships that could be construed as a potential conflict of interest.
